# Body roll amplitude and timing in backstroke swimming and their differences from front crawl at the same swimming intensities

**DOI:** 10.1038/s41598-020-80711-5

**Published:** 2021-01-12

**Authors:** Tomohiro Gonjo, Ricardo J. Fernandes, João Paulo Vilas-Boas, Ross Sanders

**Affiliations:** 1 Department of Physical Performance, Norwegian School of Sport Sciences, Oslo, Norway; 2 Institute for Sport, Physical Education and Health Sciences, The University of Edinburgh, Edinburgh, Scotland, UK; 3 Faculty of Sport, CIFI2D, and LABIOMEP, University of Porto, Porto, Portugal; 4 Faculty of Medicine and Health, The University of Sydney, Sydney, NSW Australia

**Keywords:** Fluid dynamics, Biomechanics, Musculoskeletal system

## Abstract

The current study investigated body roll amplitude and timing of its peak in backstroke and compared them with front crawl swimming. Nineteen anatomical landmarks were digitised using 80 swimming trial videos (ten swimmers × two techniques × four intensities) recorded by two above- and four below-water cameras. One upper-limb cycle was analysed for each trial, and shoulder and hip roll, whole-body roll (*WBR*), and *WBR* due to the buoyant torque (*WBR*_*BT*_) were obtained. Main effects of intensity and technique on the amplitude and timing to reach the peak in those variables were assessed by two-way repeated-measures ANOVA. Swimmers decreased their *WBR*_*BT*_ amplitude with an increase in the intensity in both techniques (p ≤ 0.005). The same result was observed for the amplitude of *WBR*, shoulder roll, and hip roll only in front crawl (p ≤ 0.017). Swimmers maintained the timing of peak *WBR*_*BT*_ in both techniques, while they shifted the timing of *WBR* and hip roll peak toward the beginning of the cycle when increasing the intensity in front crawl (p ≤ 0.017). In conclusion, swimmers maintain the amplitude of *WBR*, shoulder roll, and hip roll in backstroke when the intensity increases, whereas they reduce the amplitude of all rolls in front crawl.

## Introduction

Competitive swimming events are performed using simultaneous (butterfly and breaststroke) and alternating (front crawl and backstroke) techniques. As the names suggest, swimmers propel forward by simultaneous left and right limb motions in the former techniques, whereas they utilise alternating limb motions in the latter techniques. Due to the difference in the limb motion pattern, each group of techniques has a distinct wave-like whole-body coordination pattern. Simultaneous techniques are characterised by undulatory wave motion^1–3^. On the other hand, swimmers exhibit a torsional wave pattern, associated with an angular motion of the upper body and six-beat kicking (except for some long-distance front crawl swimmers^4^), in swimming with alternating limb motion^5,6^.

The angular motion of the body that characterises the alternating techniques is often referred to as ‘body roll’ and has been of great interest among researchers. The definition of body roll in the extant literature differs depending on the research interest. For example, some studies defined it as the angular displacement of the whole body^7,8^, whereas others have described the body roll as the rotation of a line connecting the left and right shoulder and hip joints around the long axis of the trunk^6,9,10^. In other words, the term ‘body roll’ is commonly used to describe either/both the whole-body roll (*WBR*) or/and shoulder and hip roll. Despite the different definitions, *WBR*, shoulder roll, and hip roll should be strongly related. It has been reported that, in front crawl swimming, around 50–55% of shoulder roll and 68–73% of hip roll result from *WBR*, respectively^11^. It has also been suggested that front crawl *WBR* is primarily generated by the external torque due to the gravitational and buoyant force vectors^8^.

In front crawl, there is a general agreement that hip roll amplitude decreases with an increase in the swimming velocity^11,12^. On the other hand, conflicting views have been expressed with regard to the relationship between shoulder roll amplitude and swimming velocity. It has been reported that shoulder roll amplitude decreased when the velocity increased^11^; however, a recent study showed no difference in this variable between sprint and distance pace swimming^12^. Nevertheless, both studies suggested that shoulder roll relative to the hip roll (trunk twist) increases when swimming velocity increases. In contrast to the front crawl, knowledge of body roll in backstroke is currently lacking and limited to reports in conference proceedings. It has been reported that swimmers did not change their shoulder and hip roll amplitude during a 200 m backstroke trial despite changes in the swimming velocity^13^. This finding is different from a study in 200 m front crawl in which swimmers increased their hip roll amplitude throughout the trial when the velocity decreased^10^. Similar results have been reported in a previous study in which swimmers show consistent shoulder roll and hip roll amplitude at four different swimming velocities^14^.

To achieve the fastest velocity possible, it is important to maximise the proportion of energy used for the forward thrust and minimise the energy that does not contribute to propulsion. In other words, it is important to minimise the forces and torques that do not contribute to the propulsion as they would cause low propulsive efficiency. Other important factors for attaining a large velocity are the stroke frequency (*SF*) and stroke length (*SL*). Although developing *SL* is essential for a long-term velocity improvement^15,16^, *SF* plays an important role in controlling the velocity (such as a short-term increase in the velocity and maintaining the swimming velocity during a race)^17^. From these perspectives, maintaining consistent *WBR* amplitude while increasing the swimming velocity in a short period of time appears to be mechanically ineffective since a 15% decrease in the upper-limb cycle duration (i.e. increase in *SF*) with maintaining *WBR* amplitude requires swimmers to produce a 38% additional torque in non-propulsive directions^7^. In front crawl, swimmers reduce their *WBR* as well as the shoulder and hip roll amplitude when increasing the velocity, potentially to minimise the required torque and consequently maximise the energetic efficiency^11^. In backstroke, on the other hand, it could be the case that swimmers show a constant *WBR* amplitude considering the unchanged shoulder and hip roll amplitude regardless of the swimming velocity^13,14^. However, *WBR* in backstroke has not been investigated.

When discussing *WBR* in alternating swimming techniques, it is essential to consider the turning effect of the body around the long axis due to the weight and buoyant force. When one upper-limb is over the water while the other is performing propelling motion in the water, the whole-body centre of buoyancy (CB) shifts away from the whole-body centre of mass (CM). This phenomenon creates a turning effect about the longitudinal axis passing through CM (buoyant torque) that is the primary mechanism for generating *WBR* in front crawl swimming^8^. There is no evidence suggesting that buoyant torque being the primary source of *WBR* in backstroke swimming. However, since both techniques consist of alternating above- and under-water upper-limb motion, it is likely that buoyant torque also plays an important role in backstroke body roll. On the other hand, the magnitude of the buoyant torque effect on *WBR* might be dissimilar between the techniques due to different body alignment. Swimmers perform lateral upper-limb recovery motion with the underwater hand path being close to the sagittal plane of the body; in contrast, swimmers recover the upper-limb over the body close to the sagittal plane and perform lateral underwater hand motion in backstroke^18,19^. Such factors might cause a difference in the distance between CM and CB on the transverse plane and might produce a different magnitude of the effect on *WBR*.

It is also important to consider the timing of each roll reaching its peak to discuss the difference in body roll between front crawl and backstroke. It has been reported that the distance between the ankles in mediolateral direction at the beginning of the backward motion of the hand is the smallest and largest in front crawl and backstroke, respectively^19^. This would make a difference in the torque production patterns around the long axis of the body between the techniques^19^, which might also produce differences in the timing of body roll. However, neither the timing nor the magnitude of *WBR*, whole-body roll due to the buoyant torque (*WBR*_*BT*_), shoulder and hip roll have been compared between the two techniques with one exception of Gonjo et al.^20^ who nevertheless only compared the maximum amplitude and angular velocity of shoulder and hip roll at the maximal swimming velocity.

To summarise, even though body roll has been widely investigated in front crawl swimming, it is unclear whether the evidence is applicable to backstroke. Given that body roll has been discussed in relation to both performance^21,22^ and clinical perspectives^23,24^ in front crawl swimming, it is necessary to establish similarities and differences in body roll mechanisms, such as the amplitude and timing of peak roll amplitude, between the two techniques at a wide range of swimming intensities. Therefore, the purposes of the current study were to investigate the body roll amplitude and timing of its peak in backstroke swimming and compare them with that of front crawl swimming. It was hypothesised that swimmers would change the maximum amplitude of *WBR*, *WBR*_*BT*_, shoulder roll, and hip roll with increasing swimming velocity in front crawl, while this would not be the case in backstroke.

## Methods

### Participants

Ten male competitive swimmers who were specialised in front crawl (n = 4), backstroke (n = 3), and individual medley (n = 3) volunteered to participate (age 17.47 ± 1.00 years, height 1.791 ± 0.054 m, body mass 69.94 ± 6.54 kg, and best records 54.50 ± 1.23 and 60.56 ± 1.29 s in short course 100 m freestyle and back crawl, respectively). Before the testing session, they were informed about the procedure, benefits, and potential risks of the study, which were approved by the ethics committees of the University of Edinburgh as well as Porto University based on the British Association of Sport and Exercise Sciences guidelines and Declaration of Helsinki. Written informed consent was then obtained from each participant or a legal guardian for minors.

### Testing protocol

The testing consisted of four 50 m trials at different speeds for both front crawl and backstroke (a total of eight 50 m bouts). The testing speeds were approximately 83, 88, 93, and 100% of their maximum swimming velocity (*v*_*83*_, *v*_*88*_, *v*_*93*_, and *v*_*max*_) in each technique that were defined by a pilot testing. Those velocities correspond to 400, 200, 100 m, and maximum effort swimming velocity in front crawl according to a dataset in a previous study^25^. Since the testing included high-intensity swimming trials, front crawl and backstroke sessions were conducted on different days (separated by 24–48 h) to minimise the fatigue effect. The order of sessions and trials was fully randomised. The testing speed was instructed to swimmers using a visual light pacer (Pacer2, KulzerTEC, Santa Maria da Feira, Portugal) at *v*_*83*_, *v*_*88*_, and *v*_*93*_ trials, and the swimmers were requested to perform their maximal effort at the *v*_*max*_ trial. The pacer was positioned at the bottom of the pool for front crawl and attached to a stainless wire above the pool for backstroke.

**Figure 1:**
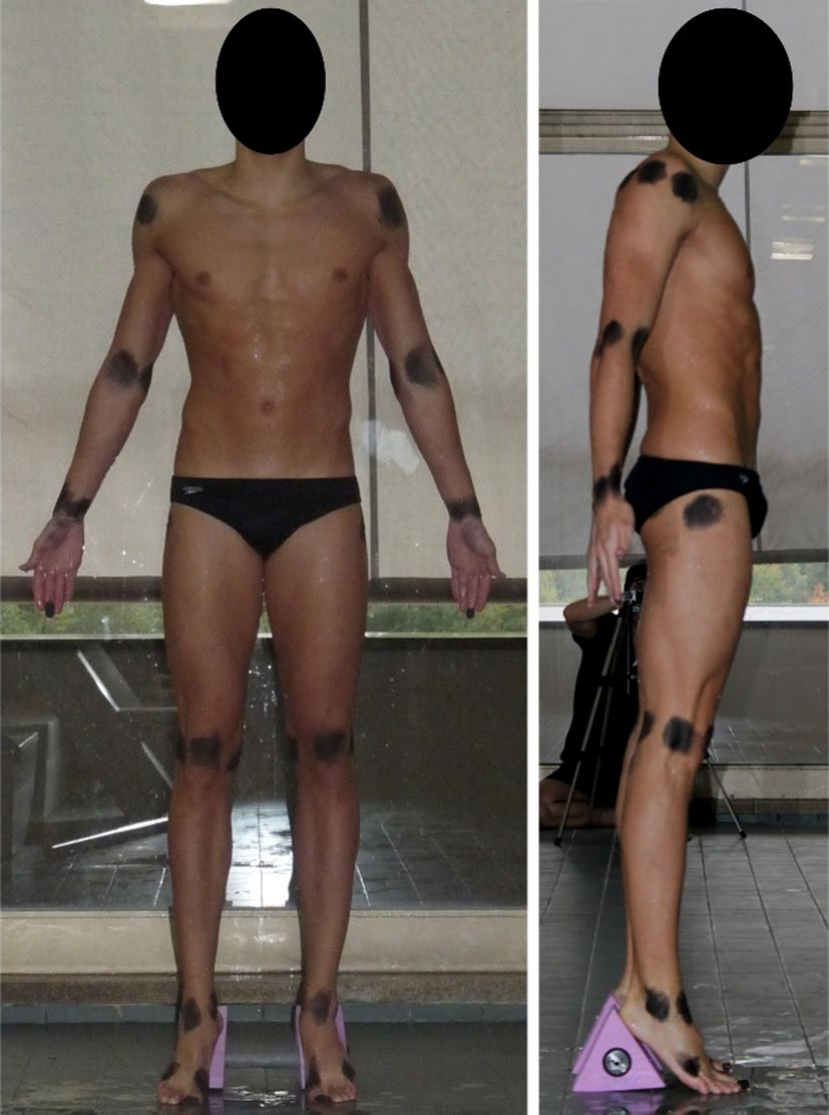
Frontal and side views of a participant marked with the oil and wax-based cream.

Participants were marked on 19 anatomical landmarks (the vertex of the head, the right and left of the: tip of the third distal phalanx of the finger, wrist axis, elbow axis, shoulder axis, hip axis, knee axis, ankle axis, fifth metatarsophalangeal joint, and the tip of the first phalanx; Fig. 1) using black oil and wax-based cream (Grimas Créme Make-Up). Each swimmer was then required to stand with the anatomical position in a calibrated space on the pool deck and photographed from front and side views simultaneously by two digital cameras (Lumix DMC-FZ40, Panasonic, Osaka, Japan). The camera images were manually digitised to apply the elliptical zone method^26^ to obtain personalised body segment parameter data. The mass, volume, CM location, and moments of inertia of each segment were obtained using the digitised data and segmental density data reported in Dempster^27^ using the ‘E-Zone’ software^28,29^.

The centre lane of a 25 m indoor pool (water and air temperature of 27 and 28 °C, respectively) was calibrated before the testing session using a calibration frame with 6 m length aligned with the swimming direction (X), 2.5 m height (Y), and 2 m width (Z)^30^ with a total of 64 control points. The control points were used as input to a three-dimensional direct linear transformation reconstruction in a later analysis, which output the reconstruction error of less than 0.1, 0.3, and 0.4% of the calibrated volume (30 m^3^) for the X-, Y-, and Z-dimension, respectively. After the calibration process, swimmers performed their individual warm-ups that were not standardised so that they were able to do an individualised session similar to their familiar competition warm-up. However, the participants were instructed to set the same warm-up intensity and distance on both testing days.

### Data collection

The calibrated space was captured by four underwater and two above-water cameras (HDR-CX160E, Sony, Tokyo, Japan), which were synchronised using a light-emitting diode system, at a sampling frequency of 50 Hz. All cameras were fixed at different heights and angles to the line of motion of the swimmer to avoid the camera axes being in the same plane. Swimmers were instructed to avoid breathing when performing front crawl since breathing motions affect their upper-limb kinematics^6,31^. Swimmers were also required to avoid underwater kicking after the push-off to ensure the calibrated space was covered entirely by their whole-body swimming motion without the influence of previous underwater locomotion. Furthermore, to minimise the influence, the latter half of 50 m was used for the analysis since some swimmers propelled longer underwater distance after the first push-off than the second one toward the calibrated space that had the same distance to both ends of the pool.

### Data processing and analysis

#### Three-dimensional coordinate data

One upper-limb cycle, defined as the period between a wrist entry to the subsequent entry of the same wrist, was selected for analysis. Analysing only one cycle was deemed adequate as competitive swimmers can produce cycle motions with a high accuracy (< 3 cm wrist path variability between cycles extracted from repeated sprint trials)^32^. The Ariel Performance Analysis System (APAS-2000 Ariel Dynamics, San Diego, CA, USA) software was used to manually digitise the 19 anatomical landmarks from all the six camera views. Every second video field was digitised, which resulted in the sampling frequency of the obtained data of 25 Hz^33^. Incorporating the direct linear transformation algorithms in APAS, three dimensional (3D) coordinates of the anatomical landmarks were obtained and smoothed using a 4th order Butterworth filter with a 4 Hz cut-off frequency. Five extra frames before and after the upper-limb cycle were digitised with additional 20–30 extrapolated points beyond the start and finish of the cycle to minimise data distortion at each side of data sets associated with filtering^34^. Using the landmark coordinate data and personalised body segment parameter, whole-body CM of swimmers were computed for each field. The 3D coordinate data were converted to 101 points representing upper-limb cycle percentiles, by Fourier transform and inverse transform, for a greater temporal resolution of the coordinate data than the original dataset. *SF* was the inverse of the time to complete one upper-limb cycle, which was multiplied by 60 to yield units of cycles per minute. Stroke length (*SL*) was the CM X-displacement during one upper-limb cycle, and the mean forward swimming velocity during the upper-limb cycle (*vx*) was computed by dividing *SL* by the cycle duration.

#### Shoulder and hip roll calculation

Shoulder roll was defined as the angle between the projection of the line joining the left and right shoulder joint centres onto the plane perpendicular to the body roll axis and the Z-axis in this plane formed by its intersection with an arbitrary horizontal plane. Similarly, hip roll was defined as the angle between the projection of the line joining the left and right hip joint centres and the horizontal reference line^20^.

#### *WBR* calculation

*WBR* was defined as the angular displacement of the entire body around the X-axis at each instant during the upper-limb cycle. *WBR* was calculated by dividing the component of the whole-body angular momentum vector representing the rotation about the X-axis by the corresponding moment of inertia at each time and integrating it over the cycle^7,8^. The calculated *WBR* angle was adjusted so that the mean angle became 0°. This adjustment was reasonable under the assumption that the analysed upper-limb cycle represented the swimmer’s typical cycle motion; otherwise, the body alignment of swimmers would change in every cycle. The angular momentum (*H*) of the whole body was calculated as the sum of local and remote angular momenta of the body segments^19,35^. The remote angular momentum (*HR*) was calculated as:

$${HR}_{si}=\frac{{m}_{s}\bullet ({v}_{s(i-1)})\times ({v}_{s(i+1)})}{{t}_{(i+1)}- {t}_{(i-1)}}$$

where *HR*_*si*_ is *HR* of segment *s* at *i*th upper-limb cycle time (*t*) and *v*_*s*_ is the vector pointing to the segment CM from the whole-body CM. The local angular momentum (*HL*) of each segment was determined using:

$${HL}_{si}={I}_{si}{\omega }_{si}$$

where *HL*_*si*_ is *HL* of segment *s* about its transverse axis at the time *i*, *I*_*si*_ is the moment of inertia of segment *s* about its transverse axis that was obtained by the e-Zone software, and *ω*_*si*_ is the angular velocity vector of segment *s* at time *i*. *H* of the body segments around their long axis was assumed to be negligible, except for the trunk^19^.

When calculating *H*, the head and neck were considered as a single segment. The trunk *H* around its transverse axis was computed using the same procedure described above. However, for this segment, *H* around the long axis was also computed since it is a large segment with a considerable rotation about its long axis (represented by shouder- and hip-roll). The trunk was considered as two segments consisting of the upper torso (thorax) and lower torso (abdomen) for computing *H* around the long axis because of the relative independence of shoulder- and hip-roll described in the introduction. This computation was conducted in a similar manner as the *H* calculation around the transverse axis, but using the vectors lining between the shoulder joints (upper torso) and hip joints (lower torso) as the orientation vectors. Thereafter, the total angular momentum of the trunk was obtained as the vector sum of local and transfer terms of the combined trunk segment about its instantaneous transverse axis and the local and transfer terms of the upper and lower torso about their long axes.

#### Calculation of *WBR*_*BT*_

*WBR*_*BT*_ was also computed following the procedure described in Yanai^8^, i.e. the buoyant torque was obtained from the cross product of the position vector from the CM to CB and the buoyant force acting through the CB, which was determined at each field in the upper-limb cycle. The buoyant force was computed as the volume of the body below the water surface multiplied by the specific weight of water (taken to be 9.77 kN/m^3^). The volume and the endpoint coordinates of each segment obtained by e-Zone program and digitised video, respectively, were used to calculate the wetted body volume under the assumption of each segment having a uniform density and symmetric shape.

For video fields in which some segments were partially submerged, the volume of those segments beneath the water surface was estimated by calculating the ratio of the underwater length to the total length of the segment, which was then multiplied by the segmental volume obtained by the e-zone programme. Unlike the other partially submerged segments, the thorax and abdomen cannot be modelled as a single vector because they are large segments where the rotation about the long axis cannot be ignored. Therefore, they were divided into 100 sub-vectors that rotate around the long axis of the trunk, and the underwater and total length of each sub-vector were obtained. The submerged volume of the thorax and abdomen was then computed by multiplying the segmental volume by the ratio of the sum of the underwater length to the sum of the total length of the sub-vectors. The calculated buoyant torque was integrated to obtain *H* of the whole body due to the buoyant torque (*H*_*BT*_). The obtained *H*_*BT*_ was adjusted so that the mean *H*_*BT*_ over the stroke cycle equalled the mean *H*, and *WBR*_*BT*_ was then computed using the same process described above. The CB of an object is CM for the fluid it displaces, meaning that it is equal to the centre of the wetted volume of the object in a fluid with a uniform density. Thus, the location of the CB was computed as the weighted average of the centre of volume of each wetted segment.

#### Calculation of timing of peak roll

In one upper-limb cycle, swimmers show one positive and one negative peak in time-series roll angle data. Thus, both peaks should be considered to avoid the effect of roll asymmetry on the results. Hence, the timing of peak roll in *WBR*, *WBR*_*BT*_, shoulder and hip roll was determined as the mean of the times when swimmers exhibited positive and negative peak roll, which was expressed as the relative time (% one cycle time).

#### Statistical analysis

In the current study, all data are presented as the mean and standard deviation in dot graphs with a 2nd polynomial trend line that is based on a rationale that hydrodynamic forces in streamwise direction (that directly or indirectly affect the analysed variables in the present study) increase with approximately the square of the swimming velocity^36^. The normality of data was checked using the Shapiro–Wilk test and confirmed in all variables apart from backstroke hip roll at *v*_*88*_ and *v*_*93*_. Therefore, for all statistical procedures related to these two variables, datasets were converted using the Box–Cox transformation^37^ to apply parametric testing methods.

A two-way repeated-measures ANOVA was used to assess the effect of technique (front crawl and backstroke) and intensity (*v*_*83*_, *v*_*88*_, *v*_*93*_, and *v*_*max*_) on *SF* and *SL* as well as the mean value of the left and right peak amplitude and timing of peak in *WBR*, *WBR*_*BT*_, shoulder roll, and hip roll. Sphericity of the data was checked with Mauchly’s test, and the F value was adjusted according to the Greenhouse–Geisser procedure when the assumption of sphericity was not met. The statistical significance level for the ANOVA tests was set at p < 0.05. Multiple comparisons of the analysed variables between each intensity in a single technique and between the two techniques at each testing intensity were performed with a paired-sample t-test with adjusting the alpha-level by the Holm-Bonferroni procedure. IBM SPSS Statistics 24 (IBM Corporation, Somers, NY, USA) and MATLAB R2019a (MathWorks Inc, MA, USA) for multiple comparisons, respectively.

## Results

### Horizontal velocity, stroke frequency and length

Inter-participants mean and standard deviation for *vx, SF* and *SL* in front crawl and backstroke at the four swimming intensities are exhibited in Table 1. A significant main effect of intensity was observed in all those variables (all p < 0.001), and the main effect of technique was significant in *vx* and *SF* (p < 0.001 and 0.002, respectively) that were both larger in front crawl than in backstroke. There was no interaction between intensity and technique effect in *SF* and *SL*. Front crawl exhibited faster velocity and higher *SF* at all trials than backstroke. On the other hand, *SL* was similar between the techniques at all intensities. As the intensity (and the velocity) increased, swimmers reduced the *SL* and increased *SF* in both front crawl and backstroke.

**Table 1:** Descriptive statistics and results from ANOVA test in swimming velocity, stroke frequency, and stroke length in front crawl and backstroke.

	*vx* (m/s)	*SF* (cycles/min)	*SL* (m/cycle)
**83% of *v***_***max***_			
Front crawl	1.43 (0.13)^b,c,d^	34.11 (5.01)^b,c,d^	2.54 (0.24)^b,c,d^
Backstroke	1.28 (0.10)^b,c,d,^*	30.08 (4.09)^b,c,d,^*	2.59 (0.26)^c,d^
**88% of** ***v***_***max***_			
Front crawl	1.52 (0.13)^a,c,d^	39.40 (6.94)^a,c,d^	2.35 (0.29)^a,c,d^
Backstroke	1.36 (0.09)^a,c,d,^*	32.52 (3.33)^a,c,d,^*	2.52 (0.15)^b,d^
**93% of *v***_***max***_			
Front crawl	1.60 (0.14)^a,b^	43.74 (8.76)^a,b,d^	2.25 (0.30)^a,b,d^
Backstroke	1.42 (0.09)^a,b,d,^*	37.65 (5.16)^a,b,d,^*	2.29 (0.23)^a,b,d^
***v***_***max***_			
Front crawl	1.70 (0.04)^a,b^	51.67 (6.38)^a,b,c^	2.00 (0.25)^a,b,c^
Backstroke	1.54 (0.06)^a,b,c,^*	44.81 (4.68)^a,b,c,^*	2.07 (0.17)^a,b,c^
**ANOVA**			
Technique effect	*F* = 58.88 *p* < 0.001	*F* = 142.97 *p* < 0.001	*F* = 86.34 *p* < 0.001
Intensity effect	*F* = 43.82 *p* < 0.001	*F* = 19.94 *p* = 0.002	*F* = 2.10 *p* = 0.18
Interaction	*F* = 0.28 *p* = 0.84	*F* = 1.46 *p* = 0.25	*F* = 1.19 *p* = 0.33

*vx* mean horizontal one-cycle velocity, *SF* stroke frequency, *SL* stroke length, *v*_*max*_ maximal horizontal velocity

a; b; c; and d, difference from 83% of *v*_*max*_; 88% of *v*_*max*_; 93% of *v*_*max*_; and *v*_*max*_, respectively; *Difference from front crawl at the comparable trial.

### Maximum roll amplitude and its timing

**Figure 2:**
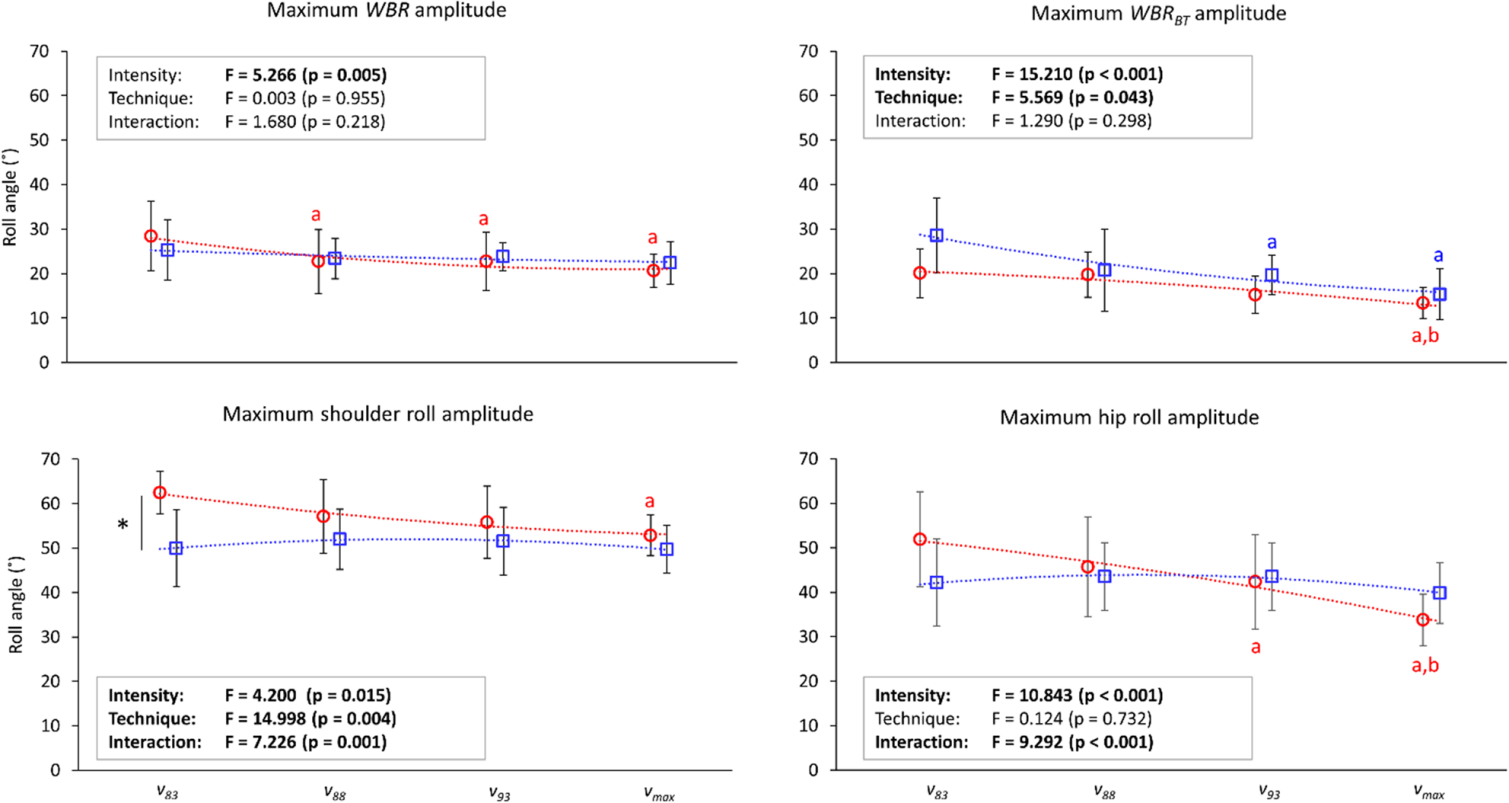
Maximum amplitudes of the whole-body roll (*WBR*), whole-body roll due to the buoyant torque (*WBR*_*BT*_), shoulder roll, and hip roll at 83, 88, 93, and 100% of the maximal velocity (*v*_*83*_*, v*_*88*_*, v*_*93*_*, and v*_*max*_, respectively). The red circles are front crawl data, red squares are backstroke data, and the dot lines represent trend lines based on the observed results. Asterisks show differences between the techniques, and a, b, and c show differences from *v*_*83*_, *v*_*88*_, and *v*_*93*_, respectively.

Maximum roll amplitude observed in the present study is shown in Fig. 2. The main effect of intensity on the roll amplitude was significant in all roll variables (p ≤ 0.015). On the other hand, the main effect of technique on the roll amplitude was only observed in *WBR*_*BT*_ and shoulder roll, in which swimmers generally showed larger amplitude in backstroke than in front crawl. The interaction between intensity and technique effect was observed in shoulder roll and hip roll.

**Figure 3:**
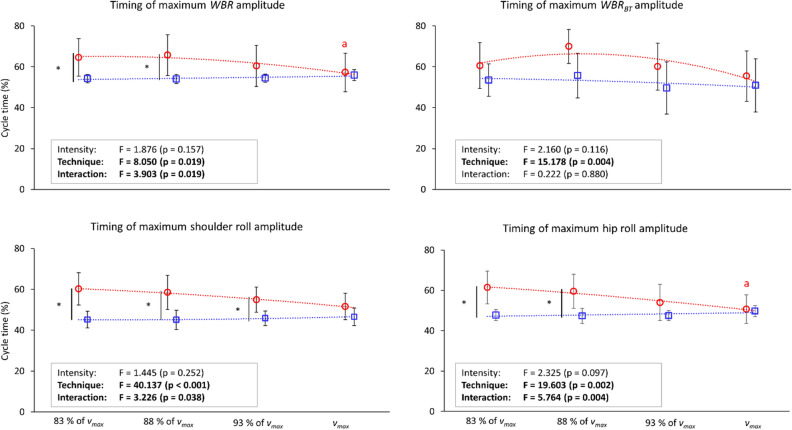
Maximum amplitude timing of the whole-body roll (*WBR*), whole-body roll due to the buoyant torque (*WBR*_*BT*_), shoulder roll, and hip roll at 83, 88, 93, and 100% of the maximal velocity (*v*_*83*_*, v*_*88*_*, v*_*93*_*, and v*_*max*_, respectively). The red circles are front crawl data, red squares are backstroke data, and the dot lines represent trend lines based on the observed results. Asterisks show differences between the techniques, and a, b, and c show differences from *v*_*83*_, *v*_*88*_, and *v*_*93*_, respectively.

A significant main effect of technique on the timing of the peak in all roll variables was observed (p ≤ 0.019), whereas the main effect of intensity on the timing of peak roll was not significant in any variables (Fig. 3). Despite the main effect, there was no difference in the timing of peak *WBR*_*BT*_ between the techniques at any intensities. The interaction between intensity and technique effect was also detected in the timing of the peak in *WBR,* shoulder roll*,* and hip roll (p ≤ 0.038). The timing of peak in *WBR*_*BT*_ and shoulder roll was earlier in backstroke than in front crawl.

Results from multiple comparisons are also shown in Figs. 2 and 3. Swimmers reduced the amplitude of rolls as the intensity increased in front crawl, but this was only observed in *WBR*_*BT*_ in backstroke. In general, differences between the techniques were particularly evident at low intensities (such as *v*_*83*_), and no difference was found in any variables related to body roll at *v*_*max*_. Front crawl had a larger value than backstroke in maximum shoulder roll amplitude and timing of maximum amplitude in *WBR*, shoulder and hip roll at *v*_*83*_. The same result was observed in timing of peak in *WBR* and hip roll at *v*_*88*_ (adjusted α-level of 0.013–0.017) and timing of shoulder roll peak at *v*_*88*_ and *v*_*93*_. Differences between trials were only observed in front crawl with a larger maximum amplitude of shoulder roll and hip roll as well as the timing of maximum amplitude of *WBR* and hip roll at *v*_*83*_ than *v*_*max*_ (adjusted α-level of 0.013–0.017).

## Discussion

The purposes of the current study were to investigate the body roll amplitude and timing of peak roll in backstroke swimming and to compare the established characteristics with those in front crawl swimming, with hypothesising that swimmers would not change their body roll amplitude in backstroke when altering the swimming velocity. Swimmers reduced the amplitude of *WBR*, shoulder roll, and hip roll with the increase in the intensity (and consequently, swimming velocity) in front crawl but it was not the case in backstroke. On the other hand, swimmers decreased *WBR*_*BT*_ when increasing the intensity in both techniques. Therefore, our hypothesis was partly supported.

Theoretically, body roll amplitude should be related to *SF*^11^; therefore, it is important to discuss observed results in *WBR*, *WBR*_*BT*_, shoulder roll, and hip roll with *SF*. In the present study, a significant main effect of swimming technique was observed in *SF* (higher in front crawl than in backstroke) without interaction between technique and intensity. On the other hand, there was no main effect of technique in *SL*. The difference in *SF* and similarity in *SL* between the techniques were clear from the multiple comparisons where the difference between the techniques was found at all trials in *SF* but not in *SL*. Thus, it is evident that front crawl is faster than backstroke due to its higher *SF* regardless of the swimming intensity. There has been no study that compared *SF* and *SL* in front crawl and backstroke swimming at the same intensities. However, two studies^25,38^ respectively reported *SF* and *SL* in front crawl and backstroke at 400, 200, 100, and 50 m speeds. Both *SF* and *SL* in front crawl and backstroke in the present study tended to be slightly smaller than the two studies by 3–6%, which can be explained by the level of the swimmers as the two previous studies tested swimmers whose personal best record was around 90% of the world record at the time, which was higher than the level of the participants in the current study (around 80% of the world record time at the time of the testing). Nevertheless, the mean absolute difference in *SF* and *SL* between the two techniques calculated using the previous studies’ data (*SF* difference, 6.1 cycles/min; *SL* difference; 0.03 m/cycle) were very similar to those obtained in the present study (*SF* difference, 6.0 cycles/min; *SL* difference; 0.07 m/cycle).

From a perspective of propulsive efficiency, *WBR* should decrease with the increase in *SF* because maintaining *WBR* amplitude while decreasing the cycle time would require swimmers produce a large amount of torque around their long axis, which does not contribute to propulsion directly^11^. From this perspective, the results in *WBR* (consistent *WBR* amplitude in backstroke and the reduction of the amplitude with the increase in the intensity in front crawl) might suggest that the way swimmers increased their *SF* was less efficient in backstroke than that in front crawl. Interestingly, however, there was no main effect of technique nor differences between the techniques at each trial in *WBR*, meaning that swimmers rolled their entire body in front crawl as large as they did in backstroke despite the shorter time period (higher *SF*). It is important to discuss whether this was due to the passive effect (i.e. due to *WBR*_*BT*_) or to the whole-body roll driven by hydrodynamic torque that was actively produced by swimmers (*WBR*_*HT*_). In the present study, it was probably the former case; even though there was a significant main effect of intensity on *WBR*_*BT*_, the multiple comparisons did not show differences between the techniques in all trials, meaning that *WBR*_*BT*_ was larger in backstroke than in front crawl overall, but the effect was trivial. Therefore, the effect of buoyant toque on the body roll was likely stronger in front crawl than in backstroke because the torque produced a similar amount of *WBR*_*BT*_ in both techniques but in a shorter duration in front crawl.

**Figure 4:**
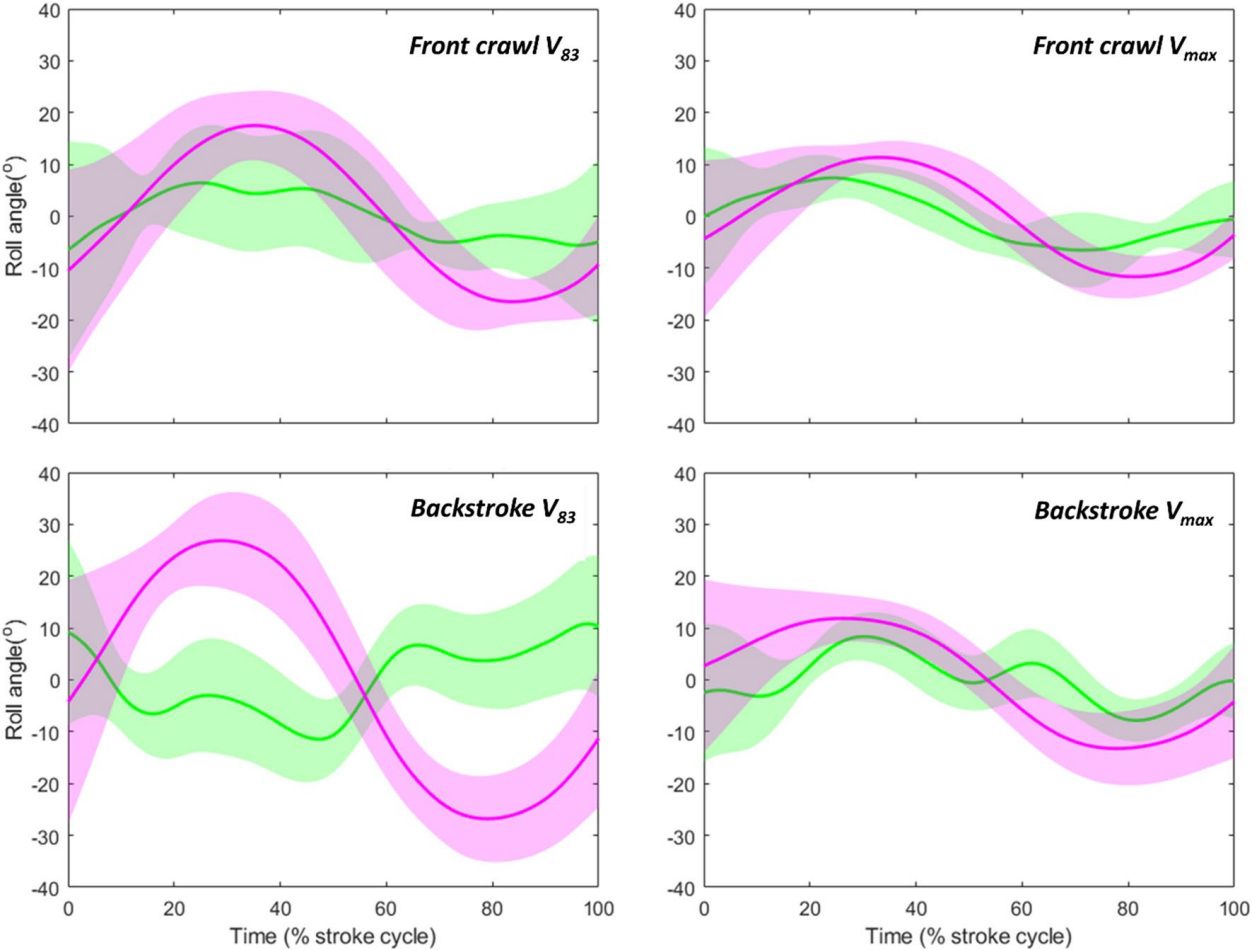
Whole-body roll angle due to the buoyant torque and due to the hydrodynamic torque (pink and green lines, respectively). The shaded areas show inter-participant standard deviation.

In front crawl, it has been reported that the primary source of *WBR* is *WBR*_*BT*_ and the amplitude of *WBR*_*BT*_ was about 79 and 59% of that of *WBR* at distance and sub-maximal paces, respectively^8^*.* In the current study, the peak amplitude of *WBR*_*BT*_ in front crawl was around 74 and 68% of the *WBR* amplitude at *v*_*83*_ and *v*_*max*_, respectively. Despite the slight differences in the absolute value, our results were in line with the previous study^8^ regarding *WBR*_*BT*_ being the primary source of *WBR*. This was also the case in backstroke in most of the trials (*v*_*88*_, *v*_*93*_, and *v*_*max*_). On the other hand, *WBR*_*BT*_ peak amplitude exceeded that of *WBR* at *v*_*83*_ in backstroke, meaning that the swimmers offset *WBR*_*BT*_ by *WBR*_*HT*_ at this intensity in backstroke. The mean *WBR*_*BT*_ and *WBR*_*HT*_ (obtained by subtracting *WBR*_*BT*_ from *WBR* at each time sample) among all swimmers in both techniques at *v*_*83*_ and *v*_*max*_ are displayed in Fig. 4. In front crawl, the timing of *WBR*_*BT*_ and *WBR*_*HT*_ peaks tended to coincide at both trials, and the same tendency was exhibited in backstroke at *v*_*max*_. On the other hand, *WBR*_*BT*_ and *WBR*_*HT*_ signals were out of phase at backstroke *v*_*83*_, which explains why the peak *WBR*_*BT*_ was about 20% larger than *WBR* at this trial in backstroke, whereas swimmers generally showed larger *WBR* than *WBR*_*BT*_ in other trials. Nevertheless, further studies focusing on the timing of different rotational effects, such as body roll wave characteristics^5^, would be required to assess how rolling action generated by different sources interact each other in front crawl and backstroke.

Swimmers reduced front crawl shoulder and hip roll amplitude with the increase in the intensity; however, they showed similar backstroke shoulder roll and hip roll amplitude at all trials. In other words, in backstroke, swimmers maintained their shoulder and hip kinematics despite the decrease in *WBR*_*BT*_. This was an interesting result since it suggested that swimmers had to roll their shoulder and hip more actively or they had to change the timing of shoulder roll and hip roll relative to *WBR*_*BT*_ when increasing their velocity. The differences in shoulder and hip roll between the techniques might be related to a characteristic of backstroke upper-limb motion. Unlike front crawl in which swimmers only perform one down- and up-sweep^39^, in backstroke, they complete more complex movements with performing down- and up-sweep motion twice during one upper-limb cycle^40^. Therefore, it might be that swimmers maintained their shoulder and hip roll despite the decrease in *WBR*_*BT*_ so that they could perform the underwater upper-limb motion with a proper body alignment; in other words, their sweeping hand might break the water surface without maintaining their shoulder and hip roll amplitude. Another possible explanation is a specific role of shoulder roll on backstroke upper-limb motion. It has been reported that hand acceleration during the first down-sweep motion occurs with shoulder roll^13^, and it has also been shown that second down-sweep completes with a maximum shoulder roll angular velocity^20^. This set of evidence might suggest that swimmers perform shoulder roll using the hydrodynamic forces produced by the second down-sweep of one hand to facilitate the entry of the other hand.

**Figure 5:**
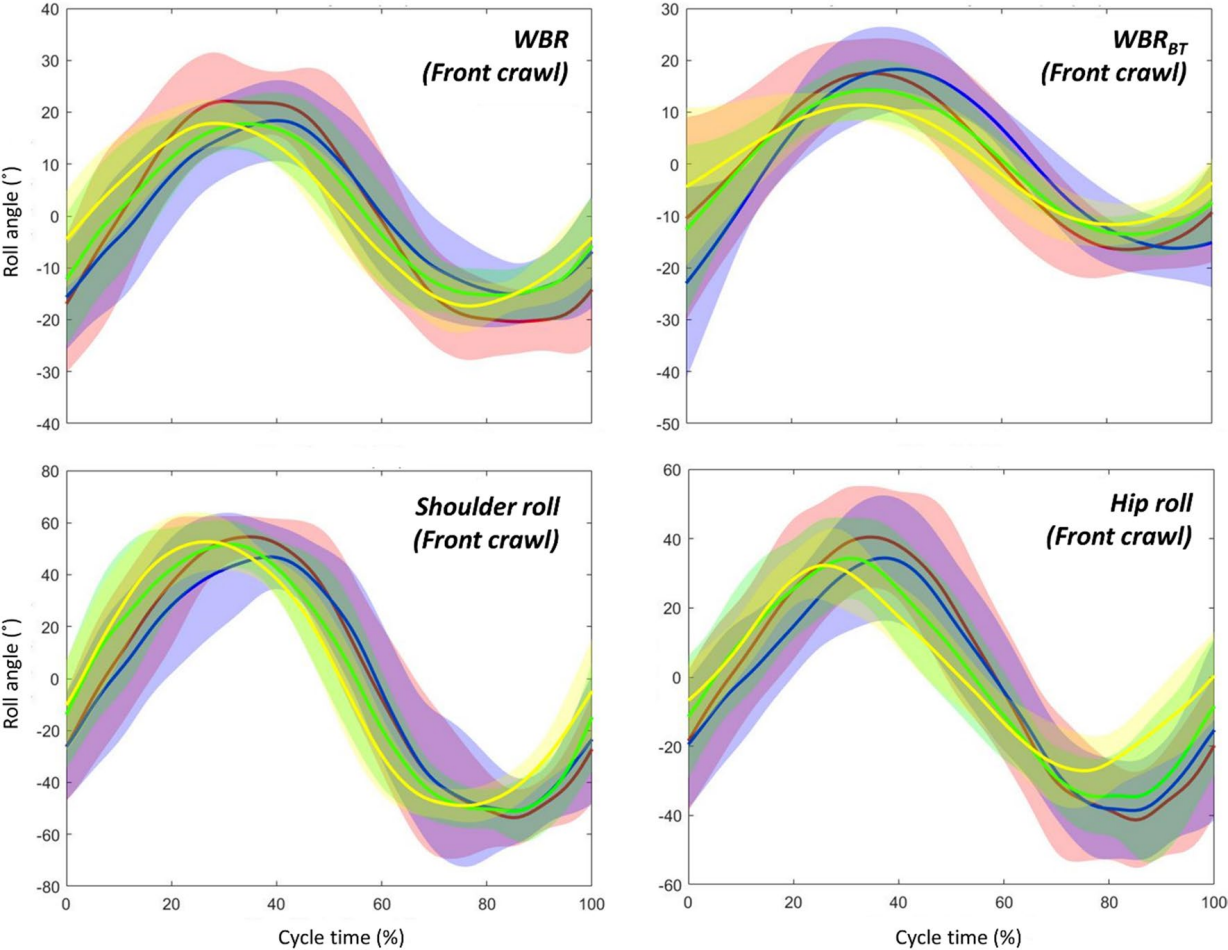
Time-series data (averaged among all participants) of the whole-body roll (*WBR*), whole-body roll due to the buoyant torque (*WBR*_*BT*_), shoulder roll, and hip roll in front crawl. Red, blue, green, and yellow lines show the averaged data at 83, 88, 93, and 100% of the maximal velocity trial, respectively, and the shaded areas shows the inter-participant standard deviation.

**Figure 6:**
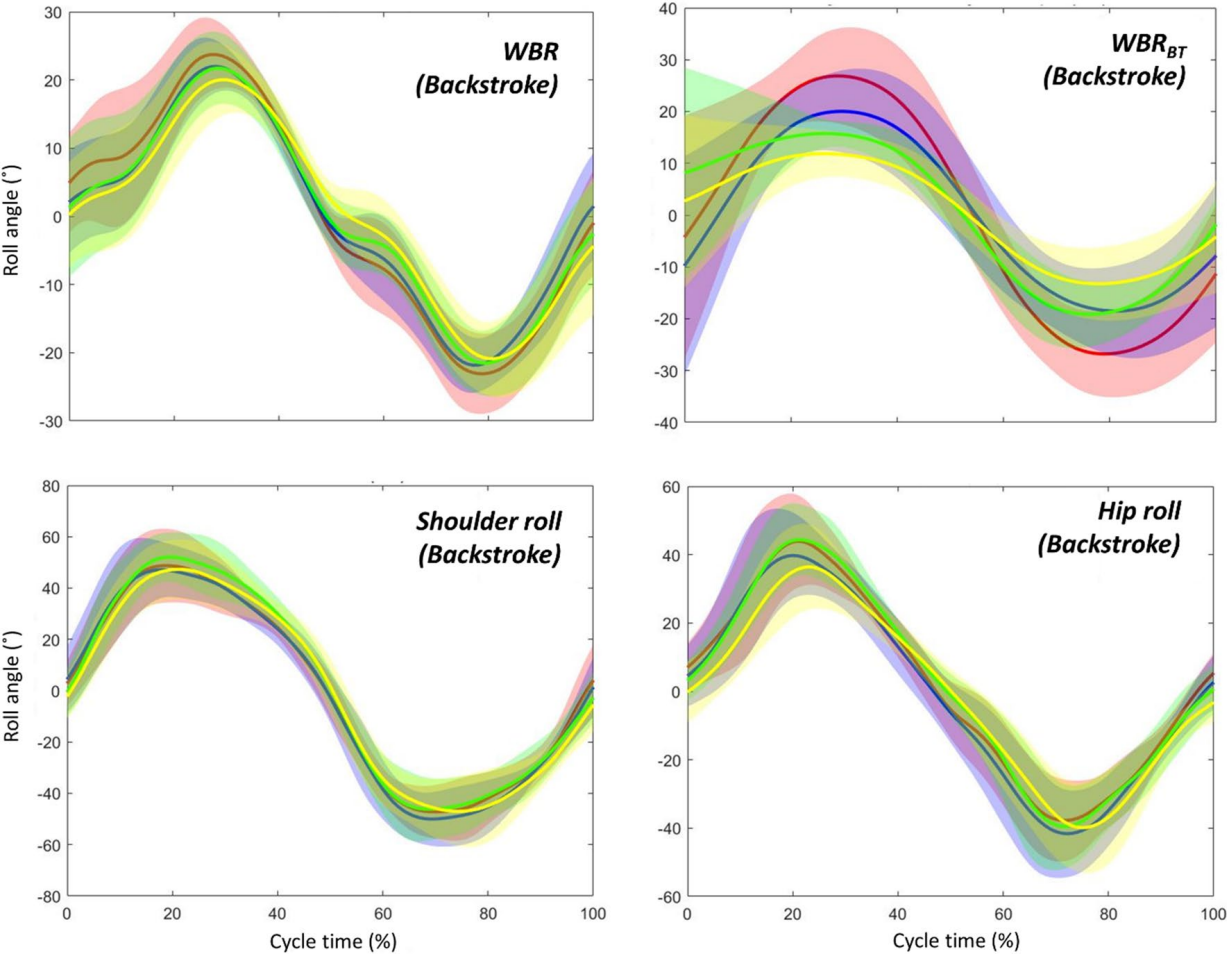
Time-series data (averaged among all participants) of the whole-body roll (*WBR*), whole-body roll due to the buoyant torque (*WBR*_*BT*_), shoulder roll, and hip roll in backstroke. Red, blue, green, and yellow lines show the averaged data at 83, 88, 93, and 100% of the maximal velocity trial, respectively, and the shaded area shows the inter-participant standard deviation.

Swimmers shifted their timing of peaks in *WBR* and hip roll to an early stage of the upper-limb cycle in front crawl as the intensity increased, whereas they showed a stable timing in backstroke regardless of the intensity. Even though the same result was not observed in shoulder roll after the adjustment of α-level due to multiple comparisons, a similar trend was exhibited in shoulder roll (Figs. 5, 6). Given that there was no main effect of intensity and interaction of it with technique effect, there are two explanations for this peak shift in front crawl. The first possibility is the swimmers changed the timing of producing hydrodynamic torque that contributed to the shift in the roll signals. The other possibility is the shift in the three roll signals was mainly due to the internal effect caused by segmental motions. In the present study, it is difficult to discuss which factors contributed to the timing shift in front crawl more, since the segmental motions and the hydrodynamic torque produced by the motion of limbs are related to each other. However, in either case, it is likely that some kinematic changes that are related to the timing of the limb motion are responsible for the shift of the three roll peaks.

**Figure 7:**
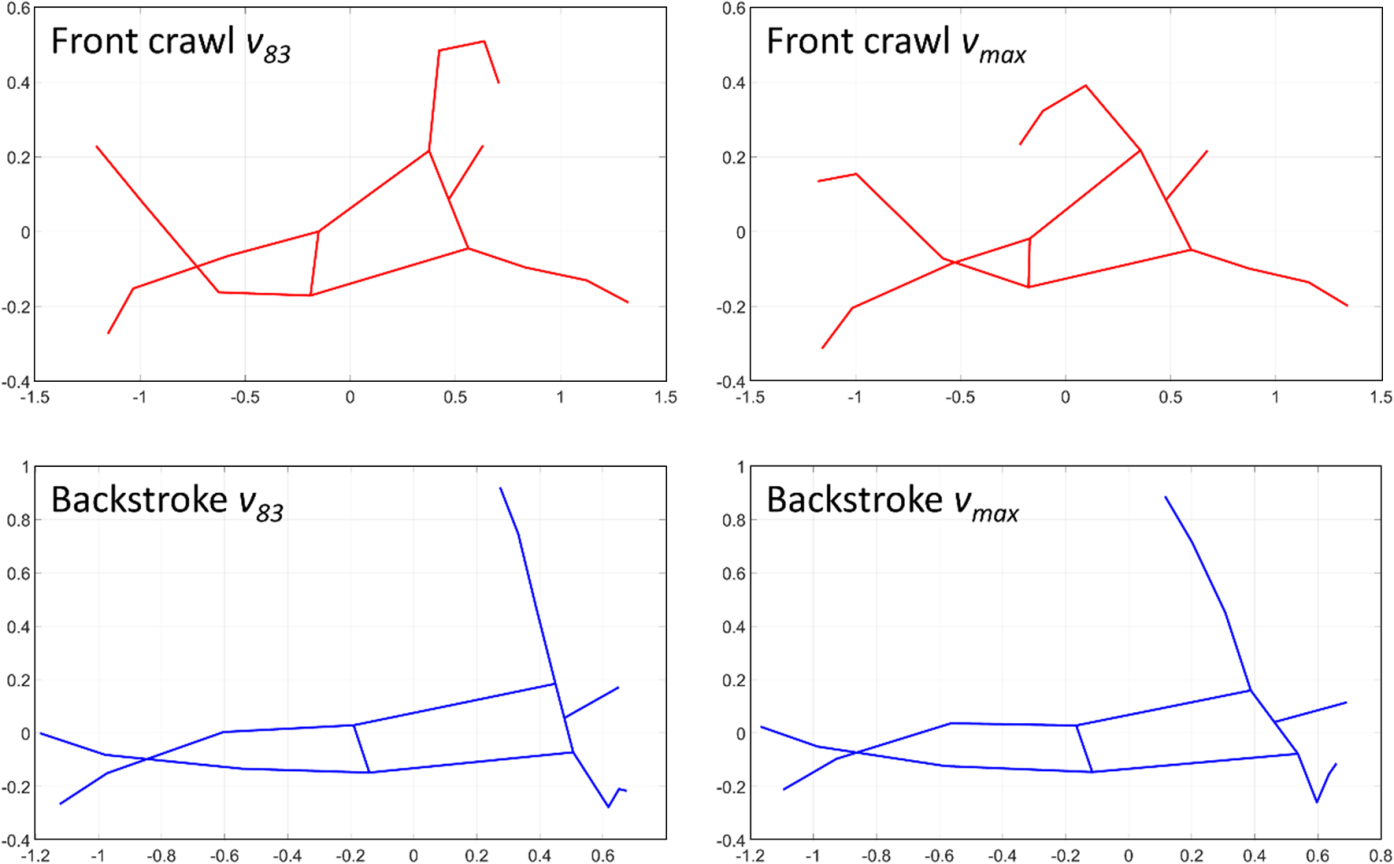
Side-view stick figure of a participant when he exhibited hip roll peak at *v*_*83*_ and *v*_*max*_.

One kinematic factor that potentially explains the shift of the peak roll timing in front crawl is inter-limb coordination. In front crawl, swimmers alter their left and right arm coordination with a change in the velocity by reducing the entry phase (from the hand entry to the water until the start of its backward motion) duration. Swimmers reduce the entry phase duration by more than 10% from a distance to a sprint pace^25^, whereas they only show a 3% change in the entry phase duration in backstroke with a corresponding pace change^38^, meaning that the transition of the coordination mode is less evident in backstroke. In sprint front crawl, the maximum shoulder roll peak is achieved when swimmers transit their motion from the entry phase to the subsequent arm pull phase^41^. This means that swimmers drive their shoulder downward throughout the entry phase, suggesting that the duration of this phase relative to the stroke cycle time should be related to the maximum shoulder roll peak amplitude and timing. Indeed, a change in the entry phase relative duration tends to match that in the shoulder roll amplitude in a 200 m front crawl^6^. As an example from the present study, Fig. 7 shows side-view stick figures of front crawl and backstroke at the time of the maximum hip roll in one swimmer. This example clearly exhibits the change in the position of upper- and lower-limbs in front crawl when the velocity was increased, whereas the limb position in backstroke was almost identical despite the change in the velocity. Nevertheless, there is currently no direct evidence supporting the relationship between the roll amplitude and the entry phase duration, which would be of interest to investigate in further studies.

The present study was the first to investigate body roll characteristics in backstroke and compared the investigated kinematics with front crawl, which provided insight into the body of alternating swimming technique knowledge. However, it should be noted that the outcomes presented in the current study should not be generalised for all populations. The buoyant torque is highly related to the anthropometry and the duration of upper-limbs being above the water in relation to the cycle time. This means that swimmers who have different anthropometric and kinematic characteristics from the participants of the current study, such as female swimmers and young swimmers, might show different results. Therefore, a wider range of groups should be tested in future studies. Furthermore, the analyses conducted in the current study contained some assumptions such as no wave around the body and the no effect of the segmental angular momentum around its long axis (apart from the trunk). Therefore, the absolute *WBR* and *WBR*_*BT*_ amplitude and timing likely contain some systematic errors. However, our focus of the study was to quantify the effect of swimming intensity on body roll and differences between front crawl and backstroke, rather than establishing the absolute *WBR* and *WBR*_*BT*_ values. As the same assumptions were applied to all trials and both techniques, the effect of the probable systematic errors on the main findings of the current study should be small.

In conclusion, swimmers decrease their *WBR*_*BT*_ with an increase in the swimming velocity in both front crawl and backstroke. It is also the case for *WBR*, shoulder roll, and hip roll in front crawl; however, swimmers maintained the amplitude of these three rolls despite the change in *WBR*_*BT*_ in backstroke. Swimmers also maintained their timing of *WBR*_*BT*_ peak in both techniques, while they shifted their timing of *WBR* and hip roll peak toward the beginning of the upper-limb cycle when increasing the velocity only in front crawl.

## References

[1] Colman V, Persyn U, Daly D, Stijnen V (1998). A comparison of the intra-cyclic velocity variation in breaststroke swimmers with flat and undulating styles. J. Sports Sci..

[2] Sanders RH, Cappaert JM, Devlin RK (1995). Wave characteristics of butterfly swimming. J. Biomech..

[3] Conceicao A, Silva AJ, Boaventura J, Marinho DA, Louro H (2013). Wave characteristics in breaststroke technique with and without snorkel use. J. Hum. Kinet..

[4] Chollet D, Chalies S, Chatard JC (2000). A new index of coordination for the crawl: Description and usefulness. Int. J. Sports Med..

[5] Sanders RH, Psycharakis SG (2009). Rolling rhythms in front crawl swimming with six-beat kick. J. Biomech..

[6] Figueiredo P, Sanders R, Gorski T, Vilas-Boas JP, Fernandes RJ (2013). Kinematic and electromyographic changes during 200 m front crawl at race pace. Int. J. Sports Med..

[7] Yanai T (2001). What causes the body to roll in front-crawl swimming?. J. Appl. Biomech..

[8] Yanai T (2004). Buoyancy is the primary source of generating bodyroll in front-crawl swimming. J. Biomech..

[9] Payton CJ, Bartlett RM, Baltzopoulos V, Coombs R (1999). Upper extremity kinematics and body roll during preferred-side breathing and breath-holding front crawl swimming. J. Sports Sci..

[10] Psycharakis SG, Sanders RH (2008). Shoulder and hip roll changes during 200-m front crawl swimming. Med. Sci. Sports Exerc..

[11] Yanai T (2003). Stroke frequency in front crawl: Its mechanical link to the fluid forces required in non-propulsive directions. J. Biomech..

[12] Andersen JT, Sinclair PJ, McCabe CB, Sanders RH (2020). Kinematic differences in shoulder roll and hip roll at different front crawl speeds in national level swimmers. J. Strength Cond. Res..

[13] Alves, F., Cardoso, L., Silva, A. & Veloso, A. Body roll and stroke kinematical changes during a race-pace swim in backstroke. In *Proceedings of the 22th International Symposium of Biomechanics in Sports.* 395–398.

[14] Gonjo, T., McCabe, C., Coleman, S. & Sanders, R. Magnitude of maximum shoulder and hip roll angles in back crawl at different swimming speeds. In *Proceedings of the 34th International Conference on Biomechanics in Sports.* 605–608.

[15] Wakayoshi K, Yoshida T, Ikuta Y, Mutoh Y, Miyashita M (1993). Adaptations to six months of aerobic swim training. Changes in velocity, stroke rate, stroke length and blood lactate. Int. J. Sports Med..

[16] Silva AF (2013). Backstroke technical characterization of 11–13 year-old swimmers. J. Sports Sci. Med..

[17] Figueiredo P, Pendergast DR, Vilas-Boas JP, Fernandes RJ (2013). Interplay of biomechanical, energetic, coordinative, and muscular factors in a 200 m front crawl swim. Biomed. Res. Int..

[18] Gonjo T (2020). Front crawl is more efficient and has smaller active drag than backstroke swimming: Kinematic and kinetic comparison between the two techniques at the same swimming speeds. Front. Bioeng. Biotechnol..

[19] Sanders RH, Gonjo T, McCabe CB (2016). Reliability of three-dimensional angular kinematics and kinetics of swimming derived from digitized video. J. Sports Sci. Med..

[20] Gonjo T, Fernandes RJ, Vilas-Boas JP, Sanders R (2020). Upper body kinematic differences between maximum front crawl and backstroke swimming. J. Biomech..

[21] Kudo S, Sakurai Y, Miwa T, Matsuda Y (2017). Relationship between shoulder roll and hand propulsion in the front crawl stroke. J. Sports Sci..

[22] Kudo S, Mastuda Y, Yanai T, Sakurai Y, Ikuta Y (2019). Contribution of upper trunk rotation to hand forward-backward movement and propulsion in front crawl strokes. Hum. Mov. Sci..

[23] Weldon EJ, Richardson AB (2001). Upper extremity overuse injuries in swimming. A discussion of swimmer's shoulder. Clin. Sports Med..

[24] Vila Dieguez O, Barden JM (2020). Body roll differences in freestyle swimming between swimmers with and without shoulder pain. Sports Biomech..

[25] Seifert L, Chollet D, Bardy BG (2004). Effect of swimming velocity on arm coordination in the front crawl: A dynamic analysis. J. Sports Sci..

[26] Jensen RK (1978). Estimation of the biomechanical properties of three body types using a photogrammetric method. J. Biomech..

[27] Dempster, W. T. Space requirements of the seated operator. *WADC Technical Report*, 55–159, (1955).

[28] Deffeyes, J. & Sanders, R. Elliptical zone body segment modeling software—digitising, modeling and body segment parameter calculation. In *Proceedings of the 23rd International Symposium on Biomechanics in Sports.* 749–752.

[29] Sanders RH (2015). Reliability of the elliptical zone method of estimating body segment parameters of swimmers. J. Sports Sci. Med..

[30] de Jesus K (2015). Reconstruction accuracy assessment of surface and underwater 3D motion analysis: A new approach. Comput. Math. Methods Med..

[31] McCabe CB, Sanders RH, Psycharakis SG (2015). Upper limb kinematic differences between breathing and non-breathing conditions in front crawl sprint swimming. J. Biomech..

[32] Sanders RH, Button C, McCabe CB (2019). Variability of upper body kinematics in a highly constrained task—sprint swimming. Eur. J. Sport Sci..

[33] Gonjo T (2019). Do swimmers conform to criterion speed during pace-controlled swimming in a 25-m pool using a visual light pacer?. Sports Biomech..

[34] Sanders R, Gonjo T, McCabe CB (2015). Reliability of three-dimensional linear kinematics and kinetics of swimming derived from digitized video at 25 and 50 Hz with 10 and 5 frame extensions to the 4th order butterworth smoothing window. J. Sports Sci. Med..

[35] Dapena J (1978). A method to determine the angular momentum of a human body about three orthogonal axes passing through its center of gravity. J. Biomech..

[36] Toussaint HM, Roos PE, Kolmogorov S (2004). The determination of drag in front crawl swimming. J. Biomech..

[37] Osborne J (2010). Improving your data transformations: Applying the Box–Cox transformation. Pract. Assess. Res. Eval..

[38] Chollet D, Seifert LM, Carter M (2008). Arm coordination in elite backstroke swimmers. J. Sports Sci..

[39] Toussaint H, Truijens M (2005). Biomechanical aspects of peak performance in human swimming. Anim. Biol..

[40] Formosa DP, Sayers MG, Burkett B (2014). Stroke-coordination and symmetry of elite backstroke swimmers using a comparison between net drag force and timing protocols. J. Sports Sci..

[41] McCabe CB, Psycharakis S, Sanders R (2011). Kinematic differences between front crawl sprint and distance swimmers at sprint pace. J. Sports Sci..

